# 3D printed liquid membrane transport system

**DOI:** 10.1016/j.ohx.2026.e00791

**Published:** 2026-05-10

**Authors:** James N. Smith, Charles F. Croft, Edward A. Nagul, Spas D. Kolev

**Affiliations:** a School of Chemistry, The University of Melbourne, Victoria 3010, Australia; b Sofia University “St. Kliment Ohridski”, Faculty of Chemistry and Pharmacy, 1 James Bourchier Blvd., Sofia, Bulgaria; c Department of Chemical Engineering, The University of Melbourne, Victoria 3010, Australia

**Keywords:** Polymer inclusion membrane, Membranes, Transport cell, Ion separation, 3D printing, Hydrometallurgy

## Abstract

In chemical separation applications, the use of membrane technologies is becoming recognised as a more environmentally friendly and cost-effective alternative to traditional systems, such as solvent extraction systems, for separating and concentrating chemicals from complex mixtures. The development of such technologies requires the use of suitable cell compartments to physically secure the membrane between the feed and receiving solutions and provide a controllable chemical environment to measure parameters such as ion flux across the membrane (a process known as ‘transport’) and selectivity for the target chemical species. However, such transport cells currently consist of expensive and often irreplaceable custom-blown glassware, in addition to custom mechanical stirring equipment due to its chemical resistance to the highly corrosive or caustic conditions of the solutions these membranes are often exposed to. The main challenges present are the design and acquisition of both this glassware and suitable mechanical stirring equipment, which impedes access to investigators studying membrane technologies who do not have access to well-funded laboratories. As an affordable and accessible solution, we have developed a 3D printed membrane transport system, analogous to traditional glassware counterparts, which incorporates polylactic acid (PLA) transport cells with a chemically resistant coating. This system is compact, simple, and yields highly reproducible results allowing for smoother workflows and better quality of data.

## Highlights

• A 3D-printed membrane transport system is described.
• The system is an affordable, accessible alternative to custom-blown glassware.
• The cell compartments tolerate up to 1 M strong base and at least 6 M strong acid.
• A commercially available magnetic stirring platform is used for solution mixing.
• The system was validated using literature conditions for Zn^2+^ transport.

## Graphical abstract

**Figure:**
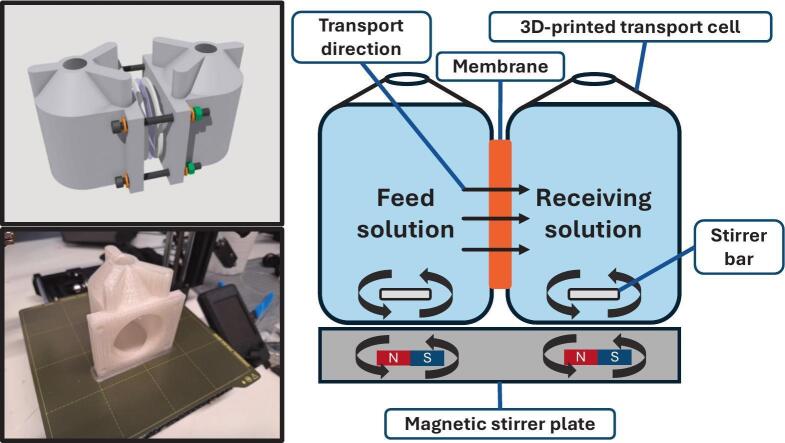

## 1 Specifications table

**Table:** 

Hardware name	*3D Printed Membrane Transport System*
Subject area	• Chemistry and biochemistry • Environmental, planetary and agricultural sciences
Hardware type	• Measuring physical properties and in-lab sensors • Other [Testing Apparatus]
Closest commercial analog	*No commercial analogue is available.*
Open source license	*CC BY-NC*
Cost of hardware	*∼ $2,200 AUD*
Source file repository	*N/A, all files available with this paper*

## 2 Hardware in context

The increasing demand for more sustainable industrial processing methods has led to a rise in membrane-based separation research, with membrane-based transport systems considered to have numerous advantages such as simultaneous extraction and back-extraction, environmental friendliness, cost-effectiveness, and simplicity of implementation [1], [2], [3], [4], [5].

Polymer inclusion membranes (PIMs), a type of liquid membrane, have attracted considerable interest in the area of membrane separation research [6], [7]. These membranes are typically fabricated using polymers as their base such as poly(vinyl chloride) (PVC), cellulose triacetate (CTA) or poly(vinylidene fluoride-*co*-hexafluoropropylene) (PVDF-HFP) [6], [7]. Extractants, which are chemical species capable of selectively separating target chemical species, are encapsulated within the polymer [8], and are usually those applied in industrial solvent extraction processes such as di-(2-ethyl hexyl) phosphoric acid (D2EHPA) or Aliquat 336 [6], [8]. Fabrication of PIMs, typically via solvent casting, is simple and effective, producing homogenous and mechanically stable membranes [6], [7]. Effective separation of chemical species for the purposes of industrial processing, chemical analysis, and sample pretreatment has been demonstrated with numerous metallic and non-metallic ionic species (e.g., rare earth elements, thiocyanate, zinc, and gold, among others [1], [2], [6], [7]). Unlike many other membrane processes, PIMs are not membrane filters and do not operate based on the physical separation of chemical species. Rather, they are dense films containing a chemical extraction reagent, often referred to as a carrier, which binds to a target chemical species and then transports it across the membrane to a receiving solution whose chemical conditions favour the dissociation of this extractant-target adduct [9], [10]. This transport occurs *via* a network of nanometre-sized channels containing the extractant and other liquid components of the PIM [8]. As such, PIMs are operated under ambient pressure, usually at room temperature, and separate the aqueous feed and receiving solutions.

**Fig. 1:**
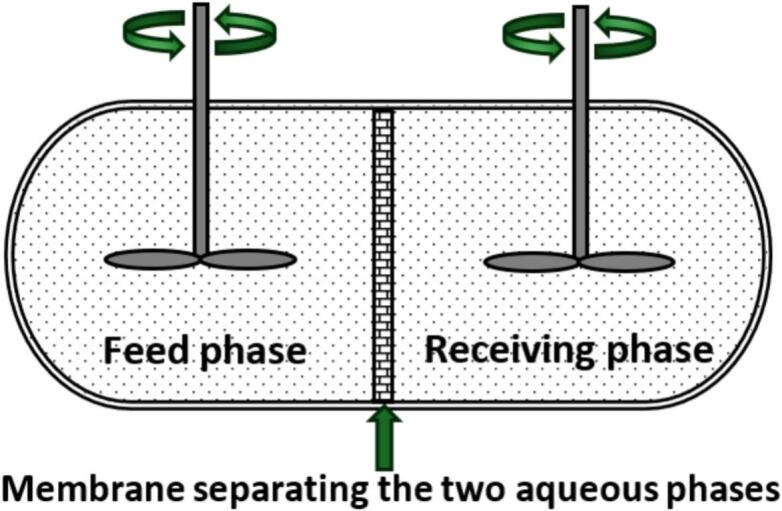
Schematic of a PIM transport cell.

A key characteristic of PIM performance is how rapidly quantitative transport of a chemical species of interest from a feed solution to a receiving solution can be achieved. This is measured by securing the PIM between a feed and receiving solution, with the transient concentration of the chemical species of interest in either solution being measured over time [7], [11], [12]. A schematic of a typical transport cell is shown in Fig. 1.

**Fig. 2:**
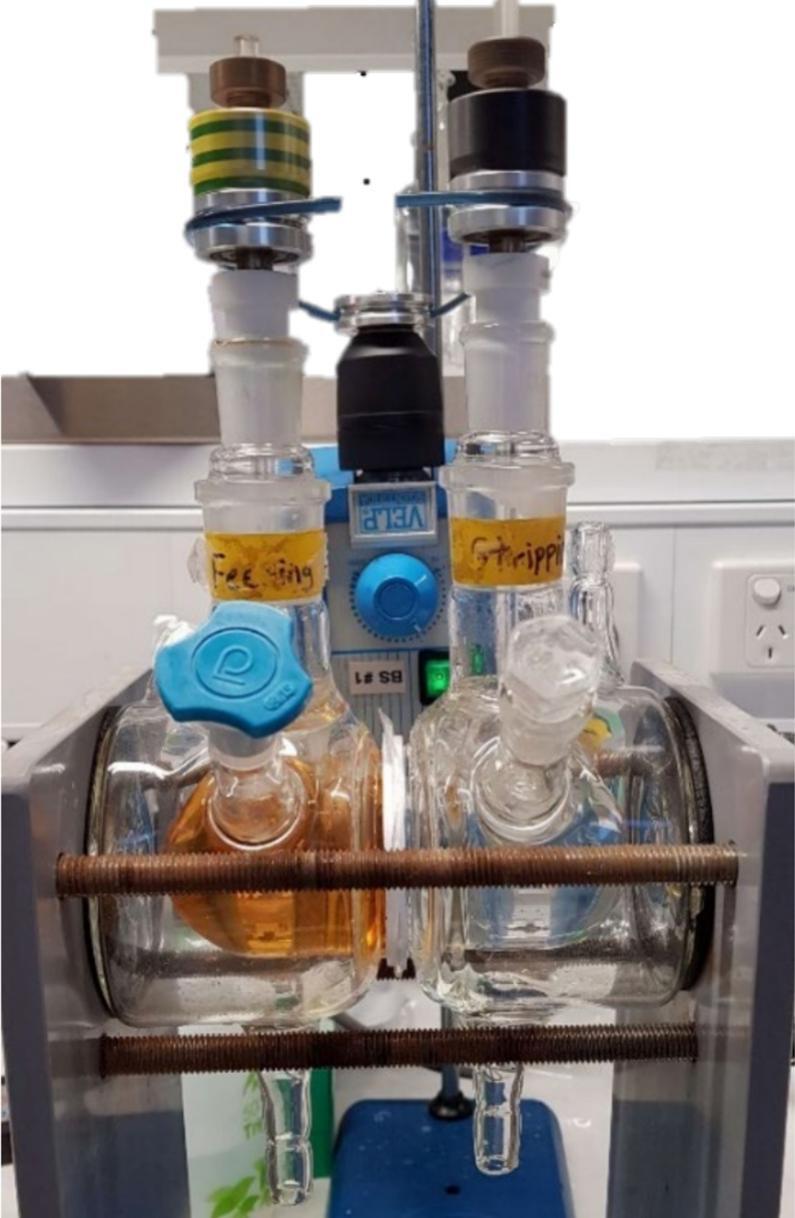
Photograph of a traditional glassware-based transport system, comprising custom-blown glass transport cells to hold the feed (left) and receiving (right) solutions, and cable-driven overhead stirrers.

Previous PIM transport studies have utilised transport cells fabricated via glass blowing, as glass is an inert and durable material [7], [11], [12]. However, fabrication of such transport cells requires access to a skilled glass blower who can produce high precision, custom laboratory-grade glassware. As such, it is often inaccessible, expensive, or non-reproducible. In addition to the glass transport cells, the corresponding transport systems require the incorporation of custom mechanical components such as overhead stirrers for solution mixing (e.g., Fig. 2). Often, these overhead stirrers are belt- or cable-driven, which can perform inconsistently between different transport systems, and can be both unreliable and difficult to set up. Furthermore, designing experiments utilising such transport systems requires testing in triplicate, as this is a minimum standard in the relevant literature for obtaining data with statistical significance. Moreover, such systems also require considerable bench space.

Recently, a novel 3D-printable transport system was developed by researchers looking for a more accessible and versatile way to develop PIMs for cadmium separation [13]. These authors similarly elaborated on the limitations of requiring custom glassware and highlighted the advantages that a simple DIY transport system can provide. Unfortunately, this system cannot be easily re-created by other researchers as the commercially-sourced components of the system were unspecified. In addition, polylactic acid (PLA) was used as the material for the test transport cells, which is not a waterproof material and is additionally unsuitable for the strongly acidic or alkaline conditions commonly used in PIM separation processes [13], [14], [15]. We found it essential to incorporate a chemically resistant and watertight spray coating which could be applied to the internal structure of the transport cell compartments developed as part of this study, thereby protecting the PLA transport cell from contact with potentially strongly acidic or alkaline solutions.

## 3 Hardware description

**Fig. 3:**
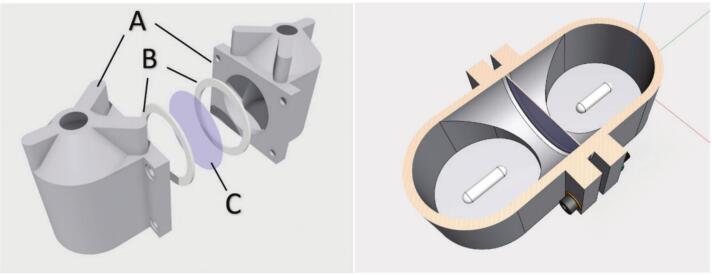
Exploded view (left) of key transport cell components showing (A) the two identical compartments, (B) PTFE gaskets, and (C) test membrane. The cross-sectional view (right) shows the internal location of the magnetic stirrer bars.

The 3D-printed transport cell incorporates two identical cell compartments with the inclusion of gaskets between them and the membrane for a watertight seal (Fig. 3). The PTFE gaskets are commercially available (ID 4.8 cm, O.D 6.4 cm, Gasketech) and are positioned on the forward faces of the cell compartments.

**Fig. 4:**
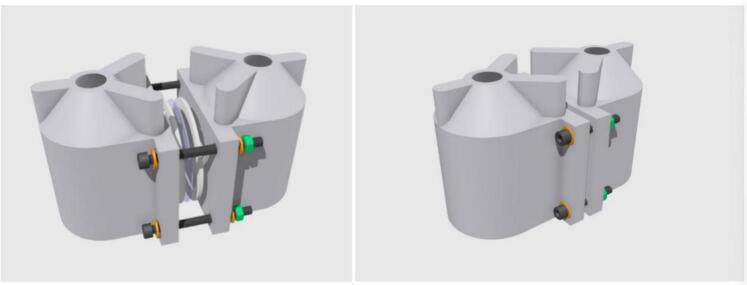
3D representations of the transport cell assembly showing all components and fastenings (left), and the fully assembled transport cell (right). M5 bolts (black) are secured with corresponding nuts (green) and washers (orange) to distribute force evenly. (For interpretation of the references to colour in this figure legend, the reader is referred to the web version of this article.)

The internal design of the cell compartments was based on the subtraction from a bulk body of two cylinders facing at a 90° degree T-intersection. The diameter of each compartment opening is 5.0 cm allowing for an exposed membrane test area of 19.6 cm^2^ and holding volume of 150 mL. These design parameters are flexible and can be modified to suit the researcher’s needs. The transport cell can easily be assembled and sealed using M5 nuts and bolts (Fig. 4). The solutions in the compartments are mixed using stirrer bars, with the transport cell placed on a 15-position VELP Scientifica magnetic stirrer platform. The compartment opening is offset 8 mm vertically from the base of the compartment to avoid collisions between the stirrer bars and the membrane.

These transport cells have been designed to fit the VELP Scientifica 15-position magnetic stirrer platform, with the cell dimensions chosen to maximize the membrane interfacial area with the solutions whilst economising space usage on the mixing platform. The volumes of the cell compartments were kept to 150 mL each, a typical solution volume for a PIM transport experiment.

Although the inclusion of the VELP mixing platform adds cost to the system, it was considered advantageous for several reasons. Firstly, the VELP mixing platform is commercially available globally through multiple suppliers. The mixing rates yielded by the platform are also highly reproducible and can reach up to 1500 rpm which is advantageous when optimising solution mixing rates. Moreover, a total of 7 transport cells can be placed on the 15-position mixing platform which overall leads to a significantly more cost-effective solution than an equivalent number of glass-blown transport cells. The transport cells also contain a sampling port on top that can be covered with a glass plate, Petri dish or 3D printed lid, allowing for visual monitoring of the PFTE magnetic stirrer bar (recommended: 25 × 7 mm cylindrical) in each cell compartment. To summarize, this transport cell setup can greatly assist with research and development (R&D) in membrane separation technology with regards to:

• Cost effectiveness and accessibility
• Versatility in chemical testing environments due to waterproof coating
• Confidence in repeatable performance between cells
• Ease of use and troubleshooting compared to traditional systems
• Economical usage of laboratory space

## 4 Design files summary

**Table:** 

Design file name	File type	Open source license	Location of the file
*Transport Cell Unit*	.STL,.STEP	*CC BY-NC*	*Available with the article*

***Transport Cell Unit –*** Main cell unit and only printable item required. File is available in .STL and .STEP formats for 3D printing and remodelling.

## 5 Bill of materials summary

**Table:** 

Designator	Component	Number	Cost per unit −currency	Total cost − currency	Source of materials	Material type
Transport Cell Unit	Cell unit	2	$2.20 AUD	$4.40 AUD	https://www.jaycar.com.au/esun-clear-pla-filament-1 kg-1–75 mm/p/TL4481	Plastic
Glass plate/watch glass		2	$5 AUD	$10 AUD	https://www.ebay.com.au/itm/182509723686	Glass
PTFE gaskets	Gasket	2	$150 (12-pack)	$150	Gasketech	Polymer
M5 x 50 mm Bolt	Bolts	4	$12 AUD	$12 AUD	https://www.ebay.com.au/itm/154641974236	Metal
M5 washer	Washer	8	$11.20 AUD	$11.20 AUD	https://www.ebay.com.au/itm/114929256931	Metal
M5 nut	Nut	4	$3.25 AUD (12-pack)	$3.25 AUD	https://www.bunnings.com.au/pinnacle-m5-stainless-steel-hex-nuts-12-pack_p0130529	Metal
Chemical resistant spray coating	Coating	1	$29.99 AUD	$29.99 AUD	https://www.ebay.com.au/itm/284984950446	Polymer
Magnetic stirrer bars	Stirrer bar	2	$13.03 AUD	$26.06 AUD	https://www.ebay.com.au/itm/166707530401	Polymer
VELP mixing platform	VELP mixing platform	1	$1953.22 AUD	$1953.22 AUD	VELP	Other: Equipment

### 5.1 Build instructions

Transport cell 3D models were generated using Shapr3D and exported in 3MF format. The models were sliced with Cura (Ultimaker; Utrecht, Netherlands) and printed using an Ender 3 V2 (Creality 3D; Shenzhen, China) during prototyping. Finalized models were printed with an Original Prusa MINI+ (Prague, Czech Republic).

**Fig. 5:**
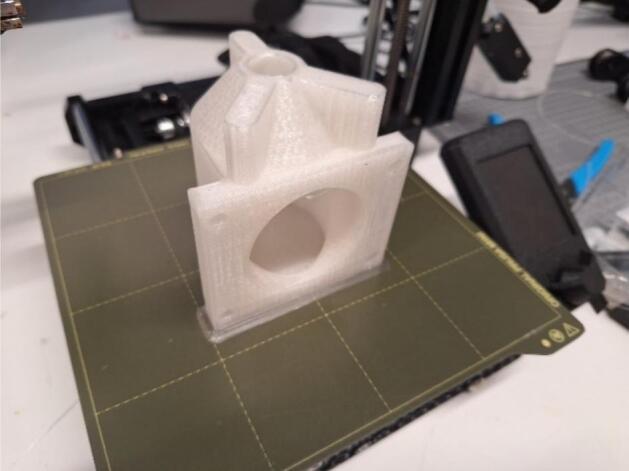
3D-printed transport cell compartment using transparent PLA+ and an Original Prusa Mini + .

The only model requiring 3D printing is the transport cell compartments, which can be fabricated in either PLA or PLA+ of any colour, since colour has no impact on their integrity. For this study, cell compartments were printed using a 0.4 mm diameter nozzle and 1.75 mm diameter PLA+ filament in either red, black, or transparent. A 0.16 mm layer height was used with an initial layer height of 0.3 mm. A high initial layer height allowed for ease of printing due to better adhesion of the filament to the base plate, and a layer height of 0.16 mm allowed for a smoother finish. A wall count of 3, in addition to 3 top and bottom layers, provided sufficient mechanical strength to the compartments whilst reducing material usage. An infill density of 30% using a triangular infill pattern provided sufficient support for the top layers when printed as shown in Fig. 5. However, the number of walls may be increased if greater mechanical strength is desired, and the infill percentage may be decreased to reduce material usage. Changes to 3D printer variables will not affect experimental results if the dimensions of the printed cell compartments are consistent. The printing and building plate temperatures were 205.0 °C and 65.0 °C, respectively, in accordance with the manufacturer’s recommendations for the filament. Printing speed was set at 50 mm/s as the default setting in Ultimaker Cura. To increase the surface quality of the print, combing mode was enabled within the infill, and Z-hop with retraction was set to only occur over printed parts at a height of 0.4 mm. The cell compartment models can be printed without supports at a fan speed of 100% starting at layer 2. To increase print adhesion to the build plate, a brim width of 8.0 mm was used to reduce warping of the plastic as it cooled, which can cause the base layer to peel off the build plate as the print proceeds.

Cells were printed individually and required approximately 8 h per build (Fig. 5). Caution should be taken during the printing process, and researchers should perform their due diligence in performing a risk assessment before operation of the machine.

**Fig. 6:**
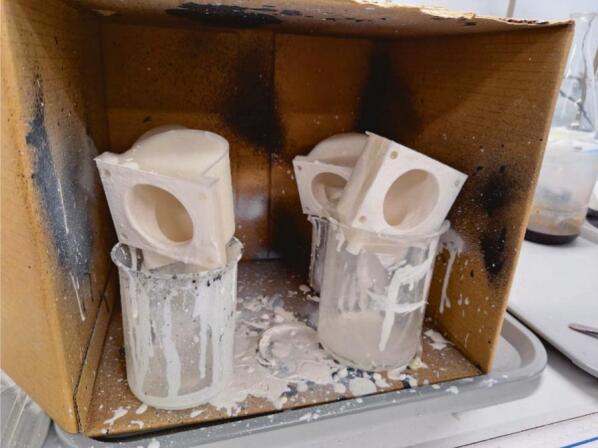
Transport cell compartments drying after application of spray coating. After coating, the cell compartments were placed upside down above beakers to allow for drainage of excess liquid.

Application of the LeakSeal spray coating should be performed in a well-ventilated area. Caution should be taken as the sealant spray is harmful to health and should only be used when proper control measures and risk assessments are in place. To waterproof the cell compartments, a uniform coating was applied to the interior and front face by rotating the compartment and by maintaining a suitable distance (instructions on spray can) of the spray from the compartment to prevent clumping of the coating. Moreover, to ensure uniform coating, cell compartments were positioned to allow excess applied coating to drip from the sampling port (Fig. 6). The manufacturer’s specifications suggest 15 – 30 min between coatings and 24 h for the coating to cure fully. A second coating was applied once dry after 2 h using the same procedure. If in doubt, the application of a third coating can be performed. It is only necessary to apply a coating to the face of the cell compartments where the gaskets will make contact, as well as the interior. Coating the full exterior of the compartments is not necessary as the internal solution is isolated from these surfaces.

Once the waterproof coating was applied, the transport cell was assembled as shown in Fig. 4 by positioning the test membrane between the two PTFE gaskets. One of the cell compartments was held upright with the membrane hole facing upwards, and the test membrane and gaskets were then carefully placed onto the face of the cell compartment. Once the gaskets were aligned with the opening, the other cell compartment was placed on top and secured with four M5 hex bolts and nuts, using washers on both sides to distribute the load.

The screws should not be excessively tightened as this could risk the plastic breaking. Only a moderate level of tightening is required and can be assessed by adding deionized water to the cell compartments and checking for leaks. If leakage occurs under the membrane, then the screws should be further tightened until this leakage ceases. Since print reproducibility may change due to the use of different filaments and/or printers, the number of walls and the infill percentage can be increased if mechanical failures occur due to excessive bolt tightening.

## 6 Operation instructions

**Fig. 7:**
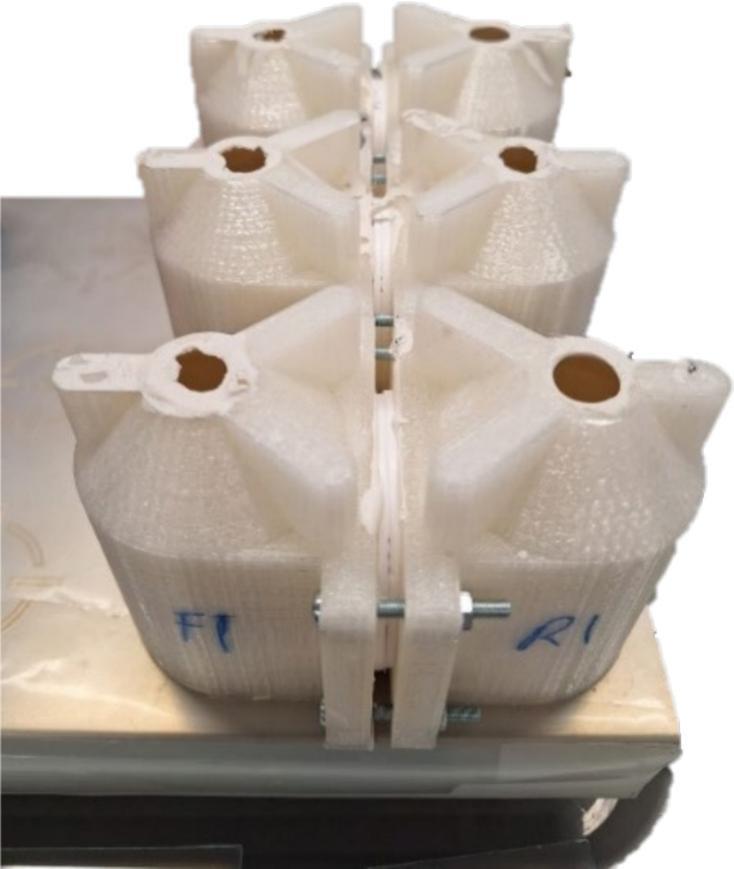
Typical setup of three transport cells positioned on the VELP magnetic stirrer platform. The compartments have been labelled F1 and R1 as the ‘feed’ and ‘receiving’ solution compartments.

Once the transport cell is assembled it can be placed onto the mixing platform, ensuring that the cell compartments line up with the magnetic stirring positions (Fig. 7). The magnetic stirrer bars should be inserted carefully via the sampling port and should align to the correct position at the bottom of the cell due to the magnetic field of the stirring platform. If the stirrer bars become attracted to each other or do not align correctly, a long thin spatula or tweezers can be used to move the bars into the correct positions.

The feed and receiving solutions should be carefully added via the sampling ports which can then be covered by a glass plate, Petri dish or 3D printable lid also coated with the chemically resistant spray. The covering of the sample ports is to both protect the solutions from contamination and help reduce evaporation during the transport process.

## 7 Validation and characterization

### 7.1 Coating stability study

Glass vials were coated with LeakSeal Flexible Rubber Coating Spray (Rust-Oleum, Australia) and left to dry in a ventilated fume cupboard for at least 72 h. Once dry, the vials were filled with potentially damaging solutions typical of those used in PIM research, i.e., strong acids or bases. Solutions were left for a minimum of 2 weeks and inspected daily for visible damage to the coating.

Initial studies investigated 1 M aqueous solutions of sulfuric acid (H_2_SO_4_, 98%, RCI Labscan), nitric acid (HNO_3_, 70%, Ajax Finechem), hydrochloric acid (HCl, 32%, Ajax Finechem), sodium hydroxide (NaOH, analytical reagent, Chem-Supply), and ammonia (NH_3_, 28%, Chem-Supply). After 2 weeks of exposure, there was no visible degradation of the rubber coating on the glass vial, no visible colour change in the coating, and no evidence of colour or solids in the acid or base solutions. Identical results were obtained over the same timeframe with 6 M solutions of H_2_SO_4_, HNO_3_, and HCl, which were tested in newly prepared vials. Finally, concentrated H_2_SO_4_, HNO_3_, and HCl (18.0 M, 15.4 M, and 10.2 M, respectively) were tested with fresh vials. When exposed to conc. H_2_SO_4_, both the coating and acidic solution instantly turned yellow which progressed to dark reddish brown over the next week. The vial exposed to conc. HNO_3_ was faintly yellow in colour after a 2-week exposure, and no degradation was visible for the vial exposed to conc. HCl. Thus, the water-resistant coating, LeakSeal, was deemed to be sufficiently chemically resistant to the solutions used in PIM transport experiments, which may involve the use of receiving solutions containing up to 6 M acid [16].

### 7.2 Transport performance validation

Validation of the 3D-printed transport cells’ performance was carried out with the transport of Zn^2+^ across PIMs containing 55 wt% PVC and 45 wt% D2EHPA, a transport system which has been used in previous studies [11], [12]. These PIMs were fabricated by measured amounts of PVC (high molecular weight, Sigma-Aldrich) and D2EHPA (97%, Sigma-Aldrich) being dissolved in tetrahydrofuran (THF, HPLC grade, Honeywell) to prepare a solution of 1 g PIM material in 10 mL THF. The solution was then cast (3.75 mL) into a glass ring (internal diameter (ID) 7.5 cm) atop a glass plate. This was then covered by filter paper and an additional glass plate and left for at least 24 h to allow for solvent evaporation. PIM thicknesses were measured on an optical stereo microscope (CH-2, Olympus, Japan) and images were captured using an attached 1.3 MP USB microscope camera (MoticCam, 1000 USB 2.0, Motic), with 10 measurements taken per PIM, processed using Motic images processing software (Motic).

After solvent evaporation, PIMs were removed, cut to discs of 6.5 cm diameter, and weighed before being placed in the transport cells. The feed cell compartment contained 150 mL feed solution of 30 mg L^−1^ Zn^2+^ (ZnCl_2_, 95%, Unilab Laboratory Reagent) which was adjusted to pH 3.0 with dilute HCl using a multiparameter laboratory benchtop pH meter (SmartCHEM-Lab, TPS) equipped with a pH probe (IJ44C, Ionode). The receiving cell compartment contained 150 mL 1.0 M HCl receiving solution, which had been standardised with 1 M NaOH which in turn was standardised by 0.2 M potassium hydrogen phthalate (99.8%, Chem-Suply), using bromothymol blue (95%, Sigma-Aldrich) as indicator.

Once feed and receiving solution compartments were filled, initial samples were taken from both compartments, stirrer bars (25 × 6 mm cylindrical) were added, and the 15-position stirring platform (VELP Scientifica) was set to 1200 rpm. This mixing rate was selected based on previous optimisation of transport experiment parameters and has been reported as a practical upper limit before cavitation occurs, making sampling inconsistent [17]. Sampling was carried out at regular intervals, taking 0.5 mL samples which were replaced by the same volume of fresh feed or receiving solution.

**Fig. 8:**
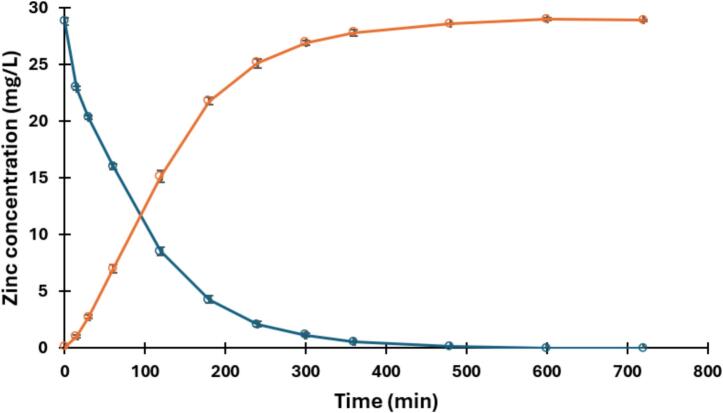
Transport of Zn^2+^ across 45 wt% D2EHPA PVC-based PIMs (thickness 42.6 ± 2.8 µm) from 30 mg L^−1^ Zn^2+^ feed solutions (•), adjusted to pH 3.0, into 1.0 M HCl receiving solutions (•). Samples of 0.5 mL were taken at predetermined time intervals and replaced with equal volumes of corresponding fresh solutions. The VELP platform was set to 1200 rpm. Error bars correspond to one standard deviation obtained from triplicate experiments.

The concentration of Zn^2+^ was measured using 1 mM Zincon monosodium salt (for spectrophotometric detection of Cu and Zn, Sigma-Aldrich) solution, which was buffered to pH 9.0 with a borate buffer. The buffer solution was made by 50 mM boric acid (99.5%, Ajax- Finechem) solution being adjusted to pH 9.0 with 1 M NaOH. For feed solution analysis, 0.5 mL of sample was mixed with 1 mL Zincon solution and 8.5 mL borate buffer. For the receiving solution, 0.5 mL of sample was mixed with 0.5 mL 1 M NaOH, 1 mL Zincon solution, and 8.0 mL borate buffer. The absorbance of the solutions was measured at 620 nm in a spectrophotometer (Libra S12, Biochrom). Calibration solutions were prepared from a Zn^2+^ stock solution (1000 ± 4 mg L^−1^, Zinc standard for AAS, Sigma-Aldrich). Measuring the Zn^2+^ concentrations in samples of the feed and receiving solutions, collected during the transport experiment, indicated that Zn^2+^ was completely transported after 600 min (Fig. 8). This result is consistent with results obtained in earlier studies of the transport of Zn^2+^ across D2EHPA-PVC-based PIMs [11], [12].

Using the VELP mixing platform, highly repeatable transport performance was obtained across three replicate transport cell experiments as demonstrated by the negligible error bars in Fig. 8. This demonstrates the effectiveness of the 3D-printed membrane transport system which, in addition to being a compact and cheap alternative to existing glass-blown cell systems, allows for new or replacement cell compartments to be printed on demand at minimal cost.

The capabilities of the 3D-printed transport cell are summarised as follows:

• Membrane interfacial surface area: 19.6 cm^2^ (5.0 cm diameter)
• Mixing rate: up to 1500 rpm
• Acid tolerance: up to 6 M strong acid
• Base tolerance: up to at least 1 M strong base
• Repeatability, as % RSD of Zn^2+^ concentrations in triplicate cell experiments (data from Fig. 8):
  o Feed: 1.7% (30 min), 4.3% (120 min), 5.7% (360 min)
  o Receiving: 6.0% (30 min), 3.5% (120 min), 0.9% (360 min)

## CRediT authorship contribution statement

**James N. Smith:** Writing – original draft, Methodology, Conceptualization. **Charles F. Croft:** Writing – review & editing, Visualization, Validation, Supervision, Investigation, Formal analysis. **Edward A. Nagul:** Writing – review & editing, Supervision. **Spas D. Kolev:** Writing – review & editing, Supervision, Resources.

## Declaration of competing interest

The authors declare that they have no known competing financial interests or personal relationships that could have appeared to influence the work reported in this paper.
